# Oral Administration of Fermented Milk from Co-Starter Containing *Lactobacillus plantarum* Y44 Shows an Ameliorating Effect on Hypertension in Spontaneously Hypertensive Rats

**DOI:** 10.3390/foods13050641

**Published:** 2024-02-20

**Authors:** Jiang Yu, Mengying Sun, Shilong Jiang, Chuqi Jiang, Guangqing Mu, Yanfeng Tuo

**Affiliations:** 1 School of Food Science and Technology, Dalian Polytechnic University, Dalian 116034, China; yujiang0324@163.com (J.Y.); smy493006403@163.com (M.S.); guangqingmu@163.com (G.M.); 2 Dalian Probiotics Function Research Key Laboratory, Dalian Polytechnic University, Dalian 116034, China; 3 Heilongjiang Feihe Dairy Co., Ltd., Beijing 100016, China; jiangshilong@feihe.com (S.J.); jiangchuqi1224@163.com (C.J.)

**Keywords:** hypertension, ACE inhibitory peptide, fermented milk, gut microbiota, SCFAs

## Abstract

Fermented dairy foods such as yogurt exhibit some beneficial effects on consumers, including relieving the symptoms of hypertension. This study aims to obtain fermented dairy products from a co-starter that have a great flavor and the auxiliary function of reducing blood pressure after longtime consumption. Commercial starter cultures composed of *Lactobacillus delbrueckii* subsp. *bulgaricus* CICC 6047 and *Streptococcus thermophilus* CICC 6038 were combined with *Lactobacillus plantarum* strains Y44, Y12, and Y16, respectively, as a combined starter culture to ferment the mixed milk of skim milk and soybean milk. The fermented milk produced using the combined starter culture mixed with *L. plantarum* Y44 showed an angiotensin-converting-enzyme (ACE) inhibitory activity (53.56 ± 0.69%). Some peptides that regulate blood pressure were released in the fermented milk, such as AMKPWIQPK, GPVRGPFPII, LNVPGEIVE, NIPPLTQTPV, and YQEPVL. In spontaneously hypertensive rat (SHR) oral-administration experiments compared with the gavage unfermented milk group, the gavage feeding of SHRs with the fermented milk produced using the combined starter culture mixed with *L. plantarum* Y44 significantly reduced the blood pressure of the SHRs after long-term intragastric administration, shown with the systolic blood pressure (SBP) and diastolic blood pressure (DBP) decreasing by 23.67 ± 2.49 mmHg and 15.22 ± 2.62 mmHg, respectively. Moreover, the abundance of short-chain fatty acids (SCFA), bacterial diversity in the gut microbiota, and SCFA levels including acetic acid, propionic acid, and butyric acid in the feces of the SHRs were increased via oral administration of the fermented milk produced using the combined starter culture containing *L. plantarum* Y44. Furthermore, the ACE-angiotensin II (Ang II)-angiotensin type 1 (AT 1) axis was downregulated, the angiotensin-converting-enzyme 2 (ACE 2)-angiotensin(1-7) (Ang1-7)-Mas receptor axis of the SHRs was upregulated, and then the RAS signal was rebalanced. The fermented milk obtained from the combined starter culture shows the potential to be a functional food with antihypertension properties.

## 1. Introduction

Hypertension is a prevalent chronic disease that stands as a leading contributor to the global disease burden. It serves as a clear risk factor for conditions such as coronary heart disease, stroke, and atherosclerosis, and causes damage to both the kidneys and cerebral blood vessels [1]. The induction of hypertension is attributed to a complex interplay of various factors, including genetics, environmental influences, vascular remodeling, excessive endothelin release, and the upregulation of the renin–angiotensin-system (RAS), among others. The RAS is a major factor controlling blood pressure changes and is mediated by the important stress hormone angiotensin II (Ang II), leading to powerful vasoconstriction effects. The heart of this system is the angiotensin-converting-enzyme (ACE), which is responsible for catalyzing the conversion of angiotensin I (Ang I) to Ang II, and it is considered to be the core enzyme of the RAS [2]. Consequently, ACE inhibitors have emerged as the primary pharmacological agents for hypertension treatment. Extracting more efficient and safer ACE inhibitors from natural food ingredients has promising prospects for the treatment of hypertension.

A growing line of the literature is based on research of the health benefits of probiotics and their fermented dairy products, such as maintaining an intestinal microecological balance, improving the intestinal barrier function, regulating the immune response, and lowering blood pressure [3,4,5]. As reported, a daily intake of some probiotic products of more than 10^9^ CFU would significantly reduce blood pressure [6]. A number of mechanisms of oxidative stress and gut microbiota can induce blood pressure regulation [7,8]. The gut microbiome is made up of several types of bacteria, fungi, archaea, viruses, and protozoa, and they are critical for maintaining host physiological homeostasis [9]. In recent years, mounds of data have shown that the gut microbiota has a major impact on host blood pressure and plays a key role in hypertension development [10]. In SHRs, microbial richness, diversity, and evenness were drastically decreased compared with normal rats [11]. Short-chain fatty acids (SCFAs), produced by gut bacteria during fermentation, have been proposed as modulators of the gut microbiota–host interaction [12]. Pluznick [13] showed in numerous experiments that new SCFA receptors are involved in blood pressure control, supporting the hypothesis that SCFAs are key mediators of the microbiota and host blood pressure regulation. These studies show that the gut microbiota could offer benefits for patients with hypertension. Notably, some lactic acid bacteria can hydrolyze the proteins in dairy products to produce short peptides with blood pressure-lowering effects [14]. For example, the short peptides Ile–Pro–Pro and Val–Pro–Pro have the same effect as antihypertensive drugs in the treatment of hypertension and play a role in lowering blood pressure by inhibiting the activities of ACEs [15]. Plant-derived beverages have many nutritional benefits and can provide essential nutrients, including vitamins, minerals, healthy fats, and fiber [16]. In addition, ACE inhibitors can also be derived from the fermentation or enzymatic breakdown of soybeans, peas, pigeon peas, chickpeas, or adzuki beans [17]. For example, the fermented mixed milk of skim milk and soy milk by lactic acid bacteria has shown improved immunomodulatory properties and the potential to prevent gastric mucosal damage [18]. Therefore, we used a combined starter culture to ferment the mixed milk of skim milk and soybean milk.

In our previous study, *L. plantarum* Y44, *L. plantarum* Y12, and *L. plantarum* Y16 exhibited high extracellular protease activities [19,20], which may lead to the release of specific bioactive peptides during milk fermentation. The intention of this study was to analyze the difference in the antihypertensive effect of fermented milk consisting of skim milk and soy milk using the commercial starter combined with *L. plantarum* strains Y44, Y12, and Y16, respectively, on spontaneously hypertensive rats (SHRs). Therefore, this study would obtain fermented milk products with a great flavor and the adjuvant effect of reducing blood pressure.

## 2. Materials and Methods

### 2.1. Materials

De Man, Rogosa, and Sharpe (MRS) broth medium was obtained from Baisi Biotechnology Co., Ltd. (Hangzhou, China). Skim milk powder was obtained from San Yuan Foods Co., Ltd. (Beijing, China). Soybeans were obtained from Ruinonghe Trading Co., Ltd. (Harbin, China). Captopril, N-benzoyl-Gly-His-Leu (HHL) and ACEs were obtained from Sigma-Aldrich Co., Ltd. (Saint Louis, MO, USA).

### 2.2. Preparation of Combined Starter Culture

*L. plantarum* Y44, *L. plantarum* Y12, and *L. plantarum* Y16 were isolated from wild turbot intestines in Dalian and stored in the Dalian Probiotics Function Research Key Laboratory with glycerin protectant at −80 °C. The commercial yogurt starter culture, consisting of *Streptococcus thermophilus* CICC 6038 and *Lactobacillus delbrueckii* subsp. *bulgaricus* CICC 6047, were obtained from the China Center of Industrial Culture Collection. The strains were subcultured three times in MRS culture medium at 37 °C for 18 h, washed with 0.85% normal saline three times, and resuspended in sterilized saline, respectively. The living cell concentrations of Y12, Y16, and Y44 strains were nearly 3 × 10^8^ CFU/mL, obtained using the plate counting method [21]. Y12, Y16, and Y44 were mixed with 6047 and 6038 bacterial suspensions at a ratio of 1:1:1 (volume ratio), respectively. The mixed bacterial suspensions were used as combined starter cultures, namely, 6047 + 6038 + Y12, 6047 + 6038 + Y16, and 6047 + 6038 + Y44.

### 2.3. Fermentation of the Mixture of Soybean Milk and Skim Milk

Soybeans were washed with ultrapure water 2–3 times to remove dust and then soaked in ultrapure water overnight. Eight times the weight of water was added to the soaked soybeans and put into the soybean milk machine for grinding for 10 min to obtain soybean milk. After that, the soybean milk was filtered with a double-filter cloth, and the residue was removed before cooling. The skim milk was prepared according to our previous study [22]. The mixed milk was made by mixing skim milk and soybean milk in a ratio of 8:2 (*v*/*v*), which was then continuously homogenized and pasteurized (95 °C, 5 min). The combined starter culture was inoculated into the pasteurized mixed milk at a ratio of 4% (*v*/*v*). The inoculated mixed milk was cultured in a constant temperature incubator at 42 °C until curding; the curdling time was about 5 h.

### 2.4. Determination of Fermented Mixed Milk Characteristics

#### 2.4.1. Titratable Acidity

The determination of titratable acidity [TA(°T)] of the fermented mixed milk samples was carried out according to the National Standards of the People’s Republic of China (GB5009.239-2016) [23].

#### 2.4.2. Viable Counts

The viable counts of lactic acid bacteria in the fermented mixed milk at the end of fermentation were determined using the pour plate method on MRS agar under an anaerobic incubation at 37 °C for 72 h, recorded as CFU/mL [21].

#### 2.4.3. Free Amino Acids

Free amino acids in the fermented milk at the end of fermentation were quantified using the *o-phthalaldehyde* (OPA) method, which was similar to our previous studies [21,24].

#### 2.4.4. Sensory Evaluation

At the end of fermentation, the sensory quality of the fermented mixed milk was evaluated according to the requirements specified in the National Standard of the People’s Republic of China (GB 19302-2010) [25]. Ten volunteers were informed consent and participated in the sensory evaluation of the fermented mixed milk. The volunteers ranged in age from 20 to 45, with five women and five men. Their daily diet include consumption yogurt 4-6 times per week. Before sensory evaluation, all fermented mixed milk samples were transferred from refrigeration at 4 °C. About 30 g of each fermented milk sample was placed in a 50 mL unscented transparent plastic cup, coded with a 3-digit random number, and equilibrated at room temperature for 30 minutes. During sensory evaluation, prepare palate-cleaning water at intervals between samples to prevent cross-over effects between samples. The criteria for the sensory evaluation of fermented milk are shown in Table A1. This experiment did not require Ethics Committee approval, because there were no risks associated for panelists who tasted samples, and this experiment met the national standards of the People’s Republic of China (GB 19302-2010) [25].

#### 2.4.5. Determination of Angiotensin-Converting-Enzyme Inhibitory (ACE I) Activity

The ACE inhibitory activity of the fermented milk samples at the end of fermentation were determined according to the method of Wu et al., with some modifications [26]. The principle is that ACE catalyzes the decomposition of the analogue of angiotensin I, hippuryl histaminoylleucine (HHL), producing hippuric acid under the conditions of 37 °C and pH 8.3, which showed a characteristic absorption peak at 228 nm. When ACEI was added, the catalytic decomposition of HHL by ACE was inhibited, and the production of hippuric acid was reduced. The analysis employed HPLC, with a Zorbax C18 column (4.6 × 250 mm, particle size 5 µm; Agilent Technologies, Santa Clara, CA, USA) at a column temperature of 30 °C and a 1 mL/min flow rate. The detection wavelength was set at 228 nm. The mobile phase consisted of acetonitrile (A) and 0.5% TFA (B) with the following elution conditions: 0–11 min, A: 20%, B: 80%; 11–12 min, A: 35%, B: 65%; 12–15 min, A: 35%, B: 65%; 16–17 min, A: 20%, B: 80%; and 18–30 min, A: 20%, B: 80%.

#### 2.4.6. Peptide Sequence Analysis

The free peptides of the fermented milk samples were determined using the method described by Mao et al. [27]. BIOPEP-UWM (https://biochemia.uwm.edu.pl/biopep-uwm, accessed on 15 April 2023) was used to screen out identified ACEIPs while combining features of ACEIP sequences to find potential ACEIPs.

### 2.5. Assay to Determine the Effects of the Fermented Milk Oral Administration on the Spontaneously Hypertensive Rats (SHRs)

Ten-week-old female SHR rats (*n* = 30) and Wistar Kyoto (WKY) rats (n = 6) were purchased from Vital River Experimental Technology Co., Ltd. (SCXK 2021-0006, Beijing, China). All animal experiments were approved by the Animal Ethics Committee of Dalian Polytechnic University (SYXK2017-0005). The rats were raised according to the method previously described in our lab [20]. After the adaptation period (7 d), thirty SHR rats were randomly divided into five groups (n = 6). After the rats were stable, they were orally administered milk at a fixed time once a day for 4 weeks. The methods of gavage are detailed in Table 1. The systolic blood pressure (SBP) and diastolic blood pressure (DBP) were monitored using the tail sleeve method (BP-2010A, Softron Beijing Biotechnology Co., Ltd. Beijing, China).

#### 2.5.1. Histological Analyses and Organ Index

After 4 weeks of gavage, all rats were weighed and euthanized. Blood samples were collected, and the serum was separated by centrifugation at 4000× *g* at 4 °C for 10 min, and frozen at −80 °C for further analysis. The kidney, heart, and liver were isolated and weighed; we soaked and fixed the entire heart in 4% paraformaldehyde fixative for the subsequent sectioning and pathological staining analysis [28]. The ImageJ2×(2.1.4.7, Rawak Software Inc.; Stuttgart, Germany) was used to analyze images. The organ index was calculated as follows: organ index = organ weight (g)/rat weight (g).

#### 2.5.2. Determination of Biochemical Indexes in Rat Serum

The levels of alanine transaminase (ALT), aspartate transaminase (AST), total cholesterol (TC), triglycerides (TG), high-density lipoprotein cholesterol (HDL-C), low-density lipoprotein cholesterol (LDL-C), superoxide dismutase (SOD), glutathione (GSH), catalase (CAT), nitric oxide (NO), and malondialdehyde (MDA) in the sera of rats from each group were determined following the instructions provided with the kits (Nanjing Jiancheng Bioengineering Institute, China). Angiotensin-converting-enzyme (ACE), angiotensin-converting-enzyme 2 (ACE 2), angiotensin 1-7 (Ang 1–7), and angiotensin II (Ang II) levels were measured according to the ELISA kit instructions (Jingmei Biotech Co., Ltd., Shenzhen, China).

#### 2.5.3. RT-PCR Analysis

We took 10 mg of kidney tissue samples from rats in each group. We crushed the samples by adding nine times the volume of 0.85% normal saline, and then centrifuged them to obtain the supernatant. The total RNA was transcribed into cDNA using the PrimeScript™ RT Master Mix kits. The mRNA expressions of ACE, ACE 2, MAS, and β-actin (with the β-actin gene serving as an endogenous control for the assay) were quantified using a StepOne Plus real-time PCR system (Applied Biosystems, Waltham, MA, USA) following the instructions provided with the ChamQ SYBR qPCR Master Mix kit [29]. The RT-reaction was as follows: the first stage was 30 s at 95 °C, the second stage was 40 cycles of 10 s at 95 °C and 30 s at 60 °C, and the third stage was 15 s at 95 °C, 60 s at 60 °C and 15 s at 90 °C.

#### 2.5.4. Fecal SCFA Analysis

We used a previously published method to prepare the fecal sample (50 mg) from the tested animals and analyzed the SCFA levels using a gas chromatography–mass spectrometry system [30].

#### 2.5.5. Rat Fecal Microbiota

All rat feces samples were forwarded to Maiwei Technology Co., Ltd. (Wuhan, China) to extract the total DNA from rat feces. The extracted DNA was analyzed using 1% agarose gel electrophoresis to ensure the appropriate amount was presented in the centrifuge tubes, and the DNA was diluted with sterile water to a concentration of 1 ng/μL. PCR was performed using the Phusion^®^ High-Fidelity PCR Master Mix with GC buffer (New England Biolabs, MA, USA), and primer targeting was in the 16S V4 region (515F GTGCCAGCMGCCGCGGTAA and 806R GGACTACHVGGGTWTCTAAT). The PCR products were analyzed using 2% agarose gel electrophoresis. The TruSeq^®^ DNA PCR-Free Sample Preparation Kit was employed to construct the library. Then, the library was quantified using Qubit and qPCR methods, followed by sequencing on the NovaSeq6000 platform. The results from each sample were processed and filtered using FLASH (V1.2.7) based on the barcode and PCR amplification primer sequences. For clustering, and the effective grouping of tags, the Uparse algorithm (Uparse v7.0.1001) was utilized with a default 97% identity threshold to group sequences into OTUs (pperational taxonomic units). The OTU sequences were annotated using the Mothur method and searching the SILVA132 SSUrRNA database (http://www.arb-silva.de/). The diversity and richness, Chao1, and Shannon calculations of samples were performed using Qiime software (Version 1.9.1).

### 2.6. Statistical Analysis

The data were expressed with the “mean ± standard deviation” of n replicates of samples. SPSS version 20.0 (SPSS, Inc., Chicago, IL, USA) software was used for mean comparisons to determine significant differences (*p* < 0.05) among the groups. The results were obtained using a one-way ANOVA. The LSD test and Dunnett’s T3 were used to analyze the significant differences when equal variances were assumed and when they were not assumed, respectively.

## 3. Results

### 3.1. Physicochemical Properties of the Fermented Mixed Milk from Combined Starter Cultures

As shown in Table 2, the mixed milk fermented with starter cultures combined with different *L. plantarum* strains exhibited different physicochemical properties. Compared with the fermented milk only produced using a commercial starter culture, the combination of the commercial starter culture with *L. plantarum* strains endowed the fermented mixed milk with different properties, particularly in terms of the ACE inhibitory activity and free amino acid contents. The ACE inhibitory activity of the fermented milk was significantly increased (*p* < 0.05) from the commercial starter mixed with *L. plantarum,* and the ACE inhibitory activity of the fermented milk produced using the combined starter culture mixed with *L. plantarum* Y44 was 55.36 ± 0.69%, significantly higher than that of the commercial starter culture fermented milk (25.98 ± 1.19%). The viable lactic acid bacteria count of fermented milk remains above 10^9^ CFU/mL and meets the national yogurt standard (GB19302-2010) [25]. Compared with the commercial starter culture fermented milk, the viable lactic acid bacteria count in the combined starter culture fermented milk was significantly higher, which may lead to the higher free amino acids, ACE inhibition activity, and TA of the fermented milk. Compared with commercial starter culture fermented milk, the combined starter culture fermented milk showed a better taste, flavor, ratio of sour to sweet, and sensory score. These results indicated that *L. plantarum* could improve the flavor of the fermented milk.

### 3.2. Analysis of Differential Peptides of the Fermented Mixed Milk from Combined Starter Cultures

**Figure 1:**
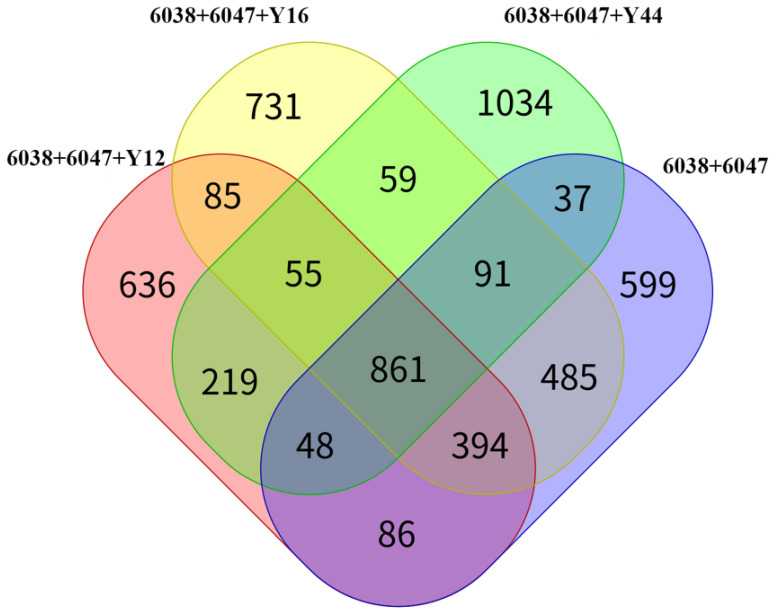
Venn diagram of differential peptides between different fermented milk groups.

The Venn diagram of the detected peptide in the fermented milk is shown in Figure 1. A total of 2561, 2384, 2404, and 2371 peptide segments were identified in samples 6038 + 6047, Y12, Y44, and Y16, respectively. Among them, 861 peptide segments were detected in all four samples, while 599, 636, 1034, and 731 peptide segments were only detected in the corresponding samples 6038 + 6047, Y12, Y44, and Y16, respectively. Among the fermented milk samples, the fermented milk from the combined starter culture with *L. plantarum* Y44 not only identified the most peptide segments, but also had the highest number of unique peptides. Out of the 2404 peptide segments, 1034 were not identified in the three other fermented milk samples, and their numbers were significantly higher than those of the three other fermented milk samples. The specific numbers of differentially expressed peptides are shown in Table 3. Whether the expression was upregulated or downregulated, there were significant differences in the numbers of peptides produced in the fermented milk using the commercial starter mixed with *L. plantarum* Y12 and *L. plantarum* Y44, compared with the commercial fermented milk. The numbers of common peptides in each of the two fermented milks were different, and the existence of these differential peptides indicated that the milk proteins in different fermented milks were decomposed by bacteria at different sites. As shown in Table 4, known ACEI peptides were screened, including AMKPWIQPK, GPVRGPFPII, LNVPGEIVE, NIPPLTQTPV, and YQEPVL, according to the BIOPEP database. We found that more types and contents of peptides were extracted from the fermented milk produced using the combined starter culture mixed with *L. plantarum* Y44, such as GPVRGPFPII (relative content of 68.8615), AMKPWIQPK (relative content of 57.9351), and NIPPLTQTPV (relative content of 76.8847). So, we inferred that the increase in the ACE inhibitory activity of the fermented milk containing *L. plantarum* 44 might be related to the type and content of ACE inhibitory peptides.

### 3.3. Effects of Fermented Milk Oral Administration on Blood Pressures of SHRs and WKY Rats

**Figure 2:**
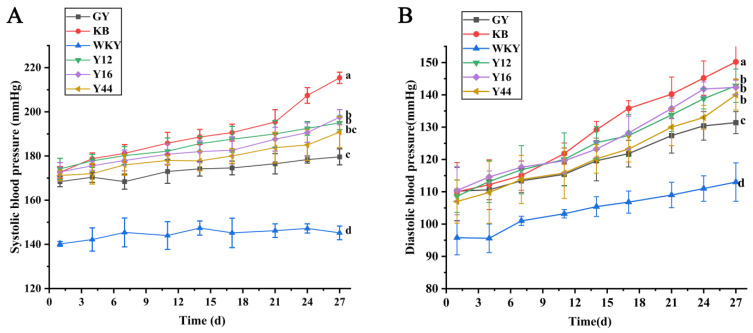
Effects of fermented milk oral administration on systolic blood pressure (SBP) (**A**) and diastolic blood pressure (DBP) (**B**) in spontaneously hypertensive rats (SHRs). Data represent the mean ± SD (*n* = 6, a–d); mean values with different letters over the bars are significantly different (*p* < 0.05) according to Duncan’s multiple range test. (GY: gavage feeding of captopril to SHRs; KB: gavage feeding of pasteurized milk to SHRs; WKY: gavage feeding of pasteurized milk to WKYs; Y12: gavage feeding of the fermented milk produced using the combined starter culture mixed with *L. plantarum* Y12 to SHRs; Y44: gavage feeding of the fermented milk produced using the combined starter culture mixed with *L. plantarum* Y44 to SHRs; Y16: gavage feeding of the fermented milk produced using the combined starter culture mixed with *L. plantarum* Y16 to SHRs).

The fermented milk showed a high ACE inhibitory activity in vitro and the presence of some peptides that could regulate blood pressure. Therefore, we investigated the antihypertensive effects of the fermented milk in the SHRs and WKY rats by monitoring the blood pressure changes after 4 weeks of oral administration of the fermented milk. As shown in Figure 2A,B, the SBP and DBP of the KB group continuously increased. Before the end of the experiment, compared with the KB group, the SBP and DBP of SHRs in the treated GY, Y44, Y12, and Y16 groups were significantly reduced (*p* < 0.05). All fermented milk by the combined starter containing *L. plantarum* alleviated elevated blood pressure, with no significant difference among the three groups. The blood pressure of the SHRs in the Y44 group showed decreased values, with an SBP and DBP of 23.67 ± 2.49 mmHg (*p* < 0.05) and 15.22 ± 2.62 mmHg (*p* < 0.05), respectively. Therefore, the long-term oral administration of the fermented milk produced using the combined starter culture with *L. plantarum* Y44 showed significant antihypertensive effects in vivo.

### 3.4. Effect of Fermented Milk Oral Administration on Renal RAS in SHRs and WKY Rats

**Figure 3:**
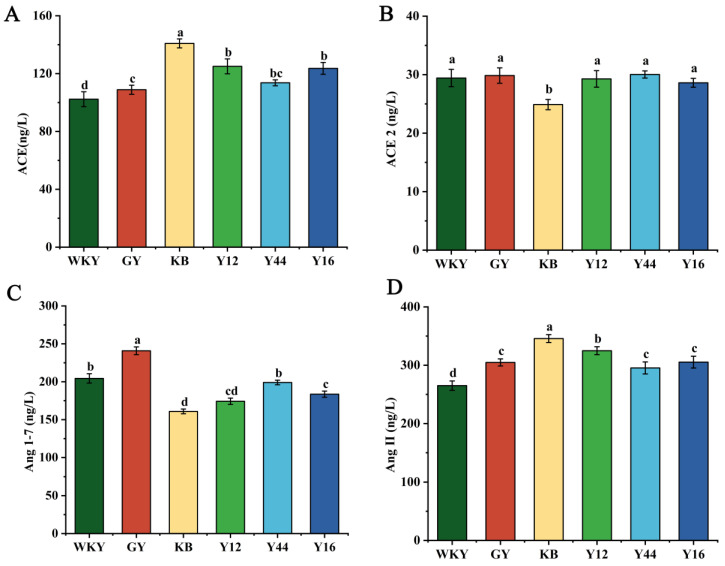
Effects of fermented milk oral administration on ACE (**A**), ACE 2 (**B**), Ang1-7 (**C**), and Ang II (**D**) in spontaneously hypertensive rats (SHRs). Data represent the mean ± SD (*n* = 6, a–d); mean values with different letters over the bars are significantly different (*p* < 0.05) according to Duncan’s multiple range test. (GY: gavage feeding of captopril to SHRs; KB: gavage feeding of pasteurized milk to SHRs; WKY: gavage feeding of pasteurized milk to WKYs; Y12: gavage feeding of the fermented milk produced using the combined starter culture mixed with *L. plantarum* Y12 to SHRs; Y44: gavage feeding of the fermented milk produced using the combined starter culture mixed with *L. plantarum* Y44 to SHRs; Y16: gavage feeding of the fermented milk produced using the combined starter culture mixed with *L. plantarum* Y16 to SHRs).

As shown in Figure 3, compared with the WKY group, the contents of ACE and Ang II in the sera of the SHRs in the KB group were significantly increased (*p* < 0.001, *p* < 0.001), while the contents of ACE 2 and Ang 1-7 were significantly decreased (*p* < 0.05, *p* < 0.01), which was in agreement with the results reported by Sun et al., who found that hypertension can affect the contents of substances related to ACE regulation in the rat sera [30]. After the long-term oral administration of the fermented milk, the contents of ACE, Ang 1-7, ACE2, and Ang II in the sera of the SHRs in the Y44 group were significantly different from those in the KB group (*p* < 0.05, *p* < 0.05, *p* < 0.05, and *p* < 0.01, respectively), while the contents of ACE, Ang 1-7, ACE 2, and Ang II in the sera of the SHRs in the GY group were significantly different from those in the KB group (*p* < 0.01, *p* < 0.05, *p* < 0.001, and *p* < 0.01, respectively). The results suggest that the fermented milk produced using the combined starter culture mixed with *L. plantarum* Y44 could rebalance RAS signaling to regulate the blood pressure by downregulating the ACE-AngII-AT1 axis and upregulating the ACE 2-Ang (1-7)-Mas axis. However, the fermented milk produced using the combined starter culture mixed with *L. plantarum* Y44 showed a lesser effectiveness than captopril in the overall regulation of the RAS.

### 3.5. Effects of Fermented Milk Oral Administration on Blood Lipid Indexes and Oxidation Indexes in the Sera of SHRs and WKY Rats

**Figure 4:**
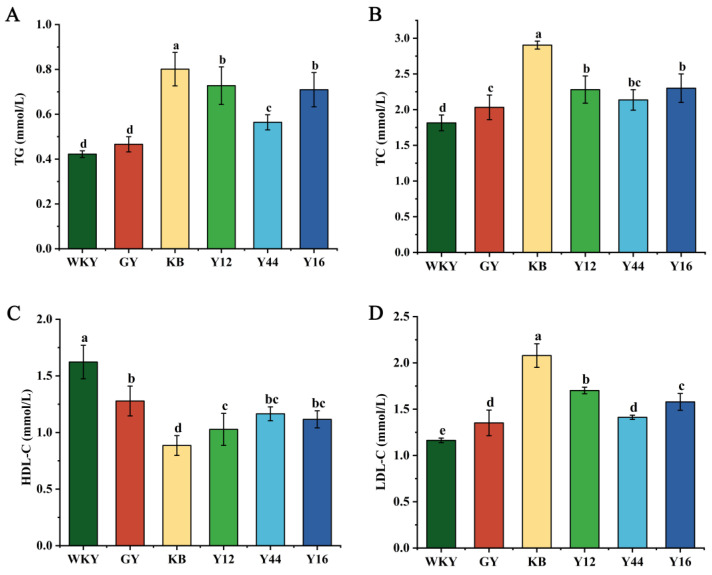
Effects of fermented milk oral administration on serum TG (**A**), TC (**B**), HDL-C (**C**), and LDL-C (**D**) in spontaneously hypertensive rats (SHRs). Data represent the mean ± SD (*n* = 6, a–d); mean values with different letters over the bars are significantly different (*p* < 0.05) according to Duncan’s multiple range test. (GY: gavage feeding of captopril to SHRs; KB: gavage feeding of pasteurized milk to SHRs; WKY: gavage feeding of pasteurized milk to WKYs; Y12: gavage feeding of the fermented milk produced using the combined starter culture mixed with *L. plantarum* Y12 to SHRs; Y44: gavage feeding of the fermented milk produced using the combined starter culture mixed with *L. plantarum* Y44 to SHRs; Y16: gavage feeding of the fermented milk produced using the combined starter culture mixed with *L. plantarum* Y16 to SHRs).

Hypertension could cause an abnormal blood lipid metabolism. As shown in Figure 4, compared with the WKY group, the contents of TG, TC, and LDL-C in the sera of the SHRs rats in the KB group were significantly increased, while the HDL-C content was significantly decreased (*p* < 0.01), which was in agreement with the results reported by Kazemi et al. [31], who found that hypertension could cause an abnormal blood lipid metabolism. Compared to the KB group, the Y44 and GY groups showed varying degrees of decrease in the TG, TC, and LDL-C contents (*p* < 0.01), but they were still higher than those in the WKY group (*p* < 0.05).

**Figure 5:**
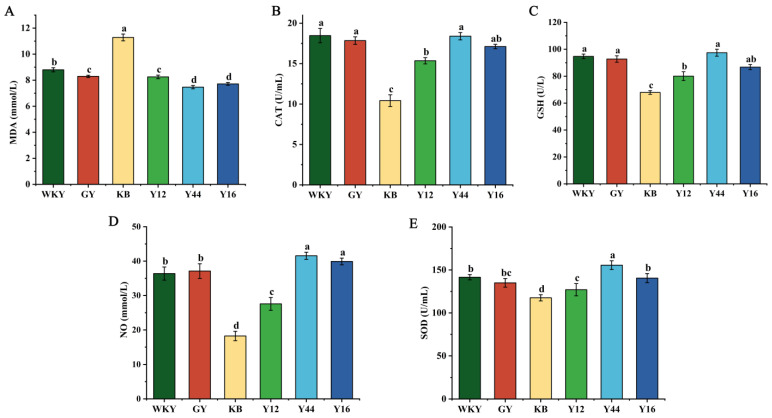
Effects of compound fermented milk on serum antioxidant enzymes in spontaneously hypertensive rats (SHRs), and the effects of MDA (**A**), CAT (**B**), GSH (**C**), NO (**D**), and SOD (**E**). Data represent the mean ± SD (*n* = 6, a–d); mean values with different letters over the bars are significantly different (*p* < 0.05) according to Duncan’s multiple range test. (GY: gavage feeding of captopril to SHRs; KB: gavage feeding of pasteurized milk to SHRs; WKY: gavage feeding of pasteurized milk to WKYs; Y12: gavage feeding of the fermented milk produced using the combined starter culture mixed with *L. plantarum* Y12 to SHRs; Y44: gavage feeding of the fermented milk produced using the combined starter culture mixed with *L. plantarum* Y44 to SHRs; Y16: gavage feeding of the fermented milk produced using the combined starter culture mixed with *L. plantarum* Y16 to SHRs).

Oxidative stress is considered a cause, consequence, or enhancing factor in the development of hypertension [32]. As shown in Figure 5, compared with the WKY group, the KB group showed significant decreases in the sera of the SHRs in terms of the antioxidant indicators (SOD, GSH, NO, and CAT, *p* < 0.05), and the content of MDA was significantly increased (*p* < 0.05). After the oral administration of the fermented milk, the MDA content was significantly decreased in the sera of the SHRs (*p* < 0.05), while the CAT activity, GSH activity, NO content, and SOD activity were significantly increased (*p* < 0.05). Among them, the oral administration of the fermented milk produced using the combined starter culture mixed with *L. plantarum* Y44 significantly decreased the content of MDA, increased the SOD activity and NO content, and relieved oxidative stress. The long-term administration of the fermented milk produced using the combined starter culture mixed with *L. plantarum* Y44 significantly relieved the increased blood pressure, oxidative stress, and hyperlipidemia in the SHRs.

### 3.6. Ameliorating Effects of Fermented Milk Oral Administration on the Liver, Heart, and Kidney Injury of SHRs and WKY Rats

**Figure 6:**
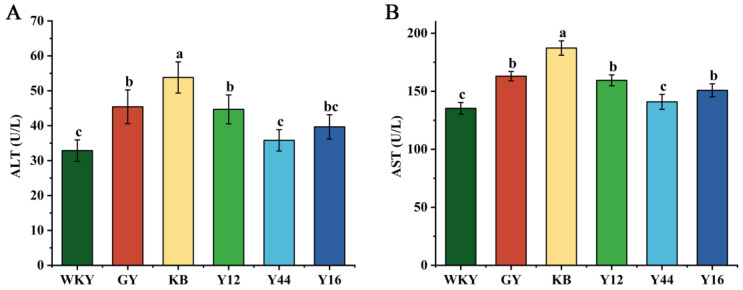
Effects of compound fermented milk on liver damage markers ALT (**A**) and AST (**B**) in sera of spontaneously hypertensive rats (SHRs). Data represent the mean ± SD (*n* = 6, a–c); mean values with different letters over the bars are significantly different (*p* < 0.05) according to Duncan’s multiple range test. (GY: gavage feeding of captopril to SHRs; KB: gavage feeding of pasteurized milk to SHRs; WKY: gavage feeding of pasteurized milk to WKYs; Y12: gavage feeding of the fermented milk produced using the combined starter culture mixed with *L. plantarum* Y12 to SHRs; Y44: gavage feeding of the fermented milk produced using the combined starter culture mixed with *L. plantarum* Y44 to SHRs; Y16: gavage feeding of the fermented milk produced using the combined starter culture mixed with *L. plantarum* Y16 to SHRs).

It is well known that an aminotransferase composed of ALT and AST is the main marker of liver injury [33]. As shown in Figure 6, compared with the WKY group, the contents of AST and ALT in the sera of the SHRs in the GY group exhibited a significant increase (*p* < 0.05), which may be related to the hepatotoxicity of captopril [34]. There was no significant difference in the contents of ATL and AST in the sera of SHRs in the Y44 group, which underwent oral administration of the fermented milk. The results indicate that captopril caused hepatotoxicity; meanwhile, the fermented milk produced by the combined starter culture mixed with *L. plantarum* Y44 was safer.

**Figure 7:**
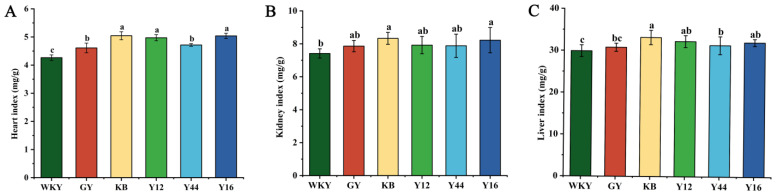
Effects of fermented milk administration on heart index (**A**), kidney index (**B**) and liver index (**C**) in spontaneously hypertensive rats (SHRs). Data represent the mean ± SD (n = 6, a–c); mean values with different letters over the bars are significantly different (*p* < 0.05) according to Duncan’s multiple range test. (GY: gavage feeding of captopril to SHRs; KB: gavage feeding of pasteurized milk to SHRs; WKY: gavage feeding of pasteurized milk to WKYs; Y12: gavage feeding of the fermented milk produced using the combined starter culture mixed with *L. plantarum* Y12 to SHRs; Y44: gavage feeding of the fermented milk produced using the combined starter culture mixed with *L. plantarum* Y44 to SHRs; Y16: gavage feeding of the fermented milk produced using the combined starter culture mixed with *L. plantarum* Y16 to SHRs).

As shown in Figure 7, compared with the WKY group, the relative weights of the kidneys, livers, and hearts of the SHRs in the KB group were significantly increased (*p* < 0.01). After the gavage treatment, the relative weights of the livers and hearts of the SHRs in the Y44 group significantly decreased (*p* < 0.05), and there was no significant difference among the other groups. This displayed that the fermented milk produced using the combined starter culture mixed with *L. plantarum* Y44 could reduce the organ damage caused by hypertension.

**Figure 8:**
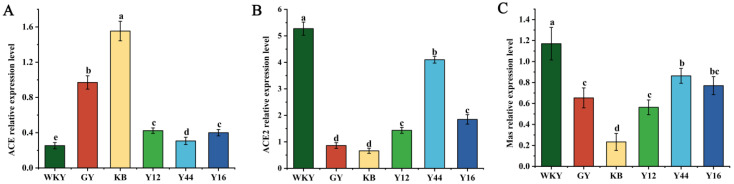
Effect of compound fermented milk on expressions of ACE mRNA (**A**), ACE 2 mRNA (**B**), and MAS mRNA (**C**) in the hearts of spontaneously hypertensive rats (SHRs).. Data represent the mean ± SD (*n* = 6, a–d); mean values with different letters over the bars are significantly different (*p* < 0.05) according to Duncan’s multiple range test. (GY: gavage feeding of captopril to SHRs; KB: gavage feeding of pasteurized milk to SHRs; WKY: gavage feeding of pasteurized milk to WKYs; Y12: gavage feeding of the fermented milk produced using the combined starter culture mixed with *L. plantarum* Y12 to SHRs; Y44: gavage feeding of the fermented milk produced using the combined starter culture mixed with *L. plantarum* Y44 to SHRs; Y16: gavage feeding of the fermented milk produced using the combined starter culture mixed with *L. plantarum* Y16 to SHRs).

As shown in Figure 8, it was observed that hypertension led to an upregulation in the ACE gene expression in the KB group (*p* < 0.001), while ACE 2 and Mas gene expressions were inhibited (*p* < 0.001). However, with the oral administration of the fermented milk produced using the combined starter culture mixed with *L. plantarum* Y44, the expression of ACE 2 and Mas genes was upregulated (*p* < 0.01). In addition, compared with the Y12 and Y16 groups, the gene expression levels of the Y44 group were more significantly upregulated. These results suggest that the fermented milk produced using the combined starter culture mixed with *L. plantarum* Y44 could reverse the imbalance between ACE and ACE 2 by downregulating the ACE-Ang II-AT1 axis and upregulating the ACE 2-Ang (1-7)-Mas axis, increasing the expression of Mas and reversing the RAS, thereby regulated blood pressure.

**Figure 9:**
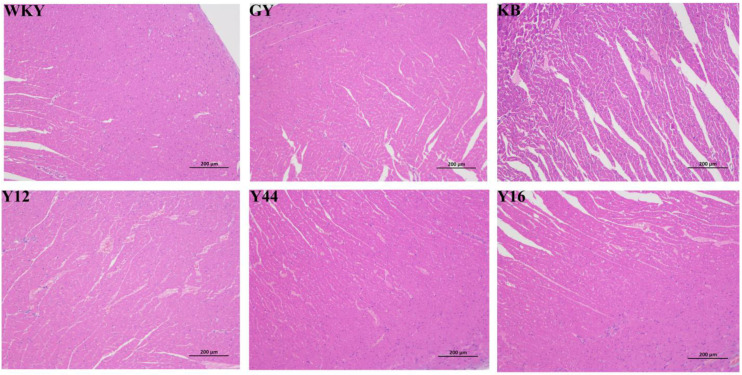
Effects of oral fermented milk on rat heart tissue (50× magnification). GY: gavage feeding of captopril to SHRs; KB: gavage feeding of pasteurized milk to SHRs; WKY: gavage feeding of pasteurized milk to WKYs; Y12: gavage feeding of the fermented milk produced using the combined starter culture mixed with *L. plantarum* Y12 to SHRs; Y44: gavage feeding of the fermented milk produced using the combined starter culture mixed with *L. plantarum* Y44 to SHRs; Y16: gavage feeding of the fermented milk produced using the combined starter culture mixed with *L. plantarum* Y16 to SHRs.

Cardiac hypertrophy in rats was primarily characterized by the enlargement of individual cardiomyocytes rather than an increase in their number. Staining techniques could be used to visually observe the morphological changes in the myocardial tissue, providing a clear representation of the damage caused by hypertension to the heart. As shown in Figure 9, the cardiomyocytes in the KB group exhibited signs of swelling and had granular contents. The cell arrangement appeared disordered, and the perinuclear space decreased in size due to myofibril extrusions. Some nuclei also displayed signs of nuclear contraction, and the area of individual cardiomyocytes increased significantly. After the intervention of the fermented milk combined with *L. plantarum* strains and captopril, some cardiomyocytes regained their normal cell shape and organized themselves in a more orderly manner. In all groups, the Y44 group demonstrated a recovery effect that was closer to the normal group (WKY). These results suggest that the fermented milk produced using the combined starter culture mixed with *L. plantarum* Y44 could effectively repair the myocardial fibrosis damage caused by hypertension.

### 3.7. Effect of Fermented Milk Oral Administration on SCFA Concentration in Rats Feces

**Figure 10:**
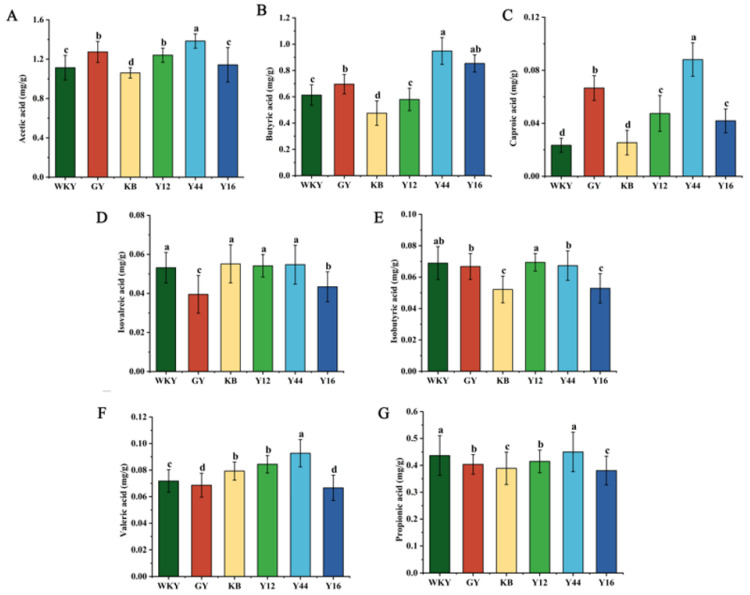
Changes of short-chain fatty acid content in the rat feces: (**A**) acetic acid, (**B**) butyric acid, (**C**) caproic acid, (**D**) isovaleric acid, (**E**) isobutyric acid, (**F**) valerate acid, and (**G**) propionic acid. Data represent the mean ± SD (*n* = 6, a–d); mean values with different letters over the bars are significantly different (*p* < 0.05) according to Duncan’s multiple range test. (GY: gavage feeding of captopril to SHRs; KB: gavage feeding of pasteurized milk to SHRs; WKY: gavage feeding of pasteurized milk to WKY rats; Y12: gavage feeding of the fermented milk produced using the combined starter culture mixed with *L. plantarum* Y12 to SHRs; Y44: gavage feeding of the fermented milk produced using the combined starter culture mixed with *L. plantarum* Y44 to SHRs; Y16: gavage feeding of the fermented milk produced using the combined starter culture mixed with *L. plantarum* Y16 to SHRs).

SCFAs are end metabolites produced from carbohydrate fermentation by gut microbes, which play an active role in regulating hypertension [35,36]. As shown in Figure 10, it could be observed that the levels of acetic acid, propionic acid, and butyric acid in the feces of rats in the KB group were significantly lower compared to the WKY group (*p* < 0.05). Compared with the KB group, the contents of acetic acid, propionic acid and butyric acid in the feces of the SHRs were significantly increased (*p* < 0.05) after the intervention of fermented milk and captopril. Among the three groups of fermented milk, the SCFA levels (acetic acid, propionic acid, and butyric acid) in the feces showed a higher value in the Y44 group than those in the Y12 and Y16 groups. These results suggest that the fermented milk produced using the combined starter culture mixed with *L. plantarum* Y44 could restore the homeostasis of SCFAs.

### 3.8. Effect of Fermented Milk Oral Administration on the Gut Microbiota of Rats

**Figure 11:**
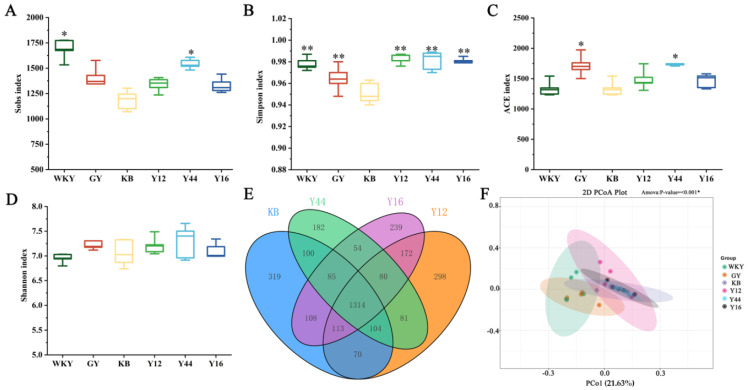
Intestinal flora diversity of rats in different treatment groups: (**A**) Sobs diversity analysis of different groups, (**B**) Simpson index diversity analysis, (**C**) ACE index analysis, (**D**) Shannon index diversity analysis, out Venn diagram outOTU number, (**E**) Venn diagram of OTU number and (**F**) PCoA analysis based on OTU. The data are expressed as the median; * *p* < 0.05, ** *p* < 0.01. (GY: gavage feeding of captopril to SHRs; KB: gavage feeding of pasteurized milk to SHRs; WKY: gavage feeding of pasteurized milk to WKY rats; Y12: gavage feeding of the fermented milk produced using the combined starter culture mixed with *L. plantarum* Y12 to SHRs; Y44: gavage feeding of the fermented milk produced using the combined starter culture mixed with *L. plantarum* Y44 to SHRs; Y16: gavage feeding of the fermented milk produced using the combined starter culture mixed with *L. plantarum* Y16 to SHRs).

As shown in Figure 11, compared with the KB group, hypertension caused significant decreases in the Sobs, ACE index, Simpson index, and Shannon index in the KB group, indicating that hypertension was associated with a reduced intestinal flora diversity. The levels of the Sobs, Simpson index, and ACE index were significantly increased (*p* < 0.05) after the oral administration of the fermented milk produced using the combined starter culture mixed with *L. plantarum* Y44. These results indicate that the fermented milk produced using the combined starter culture mixed with *L. plantarum* Y44 treatment had a significant effect on improving the diversity of intestinal flora in hypertensive rats.

**Figure 12:**
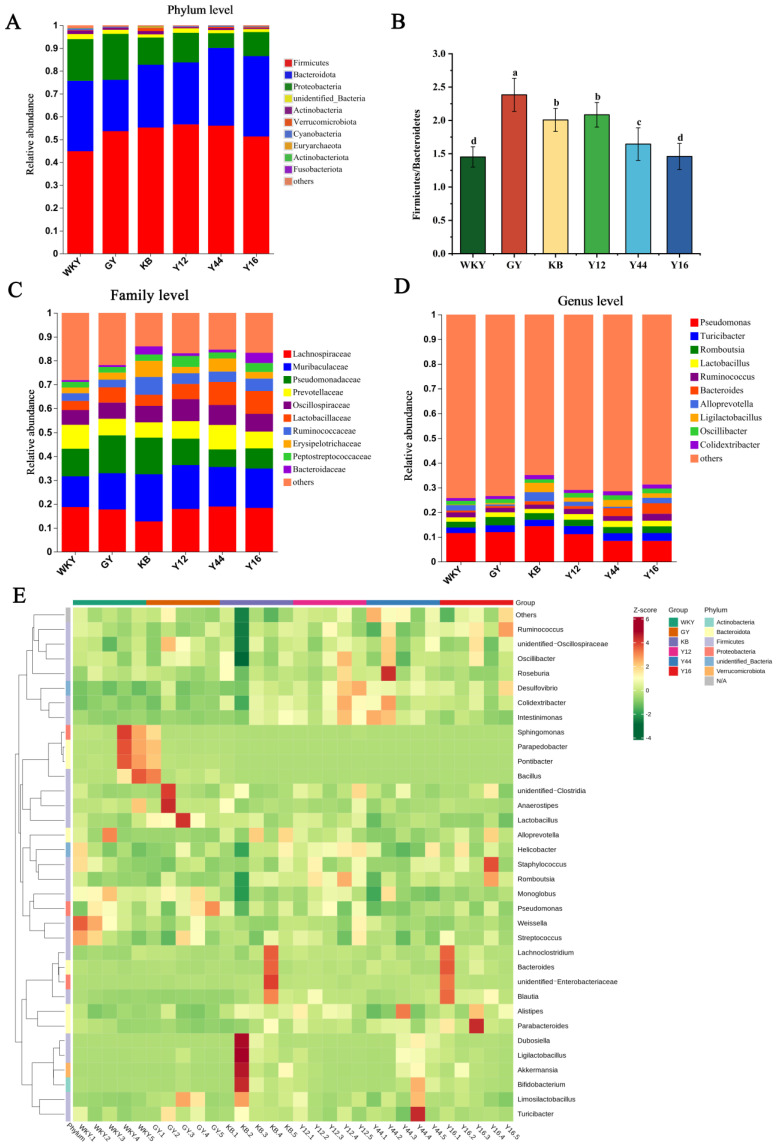
(**A**) Bar graph of gate levels for different groups, (**B**) Firmicutes/Bacteroidetes ratio for each group, (**C**) columnar distribution of intestinal flora in rats at the family level, (**D**) histogram of community structure distribution of rat intestinal flora at the genus level, and (**E**) heat map of the 30 most abundant species of bacteria in rat feces. The data in Figure 12B represent the mean ± SD (n = 6, a–d); mean values with different letters over the bars are significantly different (*p* < 0.05) according to Duncan’s multiple range test. (GY: gavage feeding of captopril to SHRs; KB: gavage feeding of pasteurized milk to SHRs; WKY: gavage feeding of pasteurized milk to WKY rats; Y12: gavage feeding of the fermented milk produced using the combined starter culture mixed with *L. plantarum* Y12 to SHRs; Y44: gavage feeding of the fermented milk produced using the combined starter culture mixed with *L. plantarum* Y44 to SHRs; Y16: gavage feeding of the fermented milk produced using the combined starter culture mixed with *L. plantarum* Y16 to SHRs).

As shown in Figure 12A,B, the dominant phyla of intestinal microorganisms were Firmicutes (F) and Bacteroides (B), accounting for 75–80%. Hypertension significantly increases the relative abundance of Firmicutes in the guts of rats, while significantly reducing the relative abundance of Bacteroides. Following the fermented milk intervention, the relative abundance of Bacteroides and Firmicutes of mice in the Y44 and Y16 groups was restored (*p* < 0.05), and the increase in the F/B ratio caused by hypertension was mitigated. As shown in Figure 12C, at the family level, compared with the WKY group, the levels of Lactobacillusceae and Lachnospiraceae were significantly decreased in the KB group, as well as the relative abundance of *Murinobacteriaceae*, *Ruminobacteriaceae*, and *Pseudomonadaceae*. The relative abundance of *Murinobacteriaceae*, Pseudomonadaceae, and *Ruminobacteriaceae* was downregulated, and the relative abundance of *Lachnospiraceae*, *Prevotellaceae*, and *Lactobacillusceae* in the SHRs was upregulated, as well as the relative abundance of rats orally administrated the fermented milk produced using the combined starter culture with *L. plantarum* Y44. As shown in Figure 12D,E, at the genus level, hypertension led to a decrease in the proportions of Turicibacter, Lactobacillus, Ruminococcus, Bacteroides, Ligilactobacillus, and Oscillibacter in the guts of SHRs in the KB group. Compared with the KB group, the oral administration of the fermented milk produced using the combined starter culture mixed with *L. plantarum* Y44 restored the relative abundance of Romboutsia, Alloprevotella, and Colidextribacter induced by hypertension, and the relative abundance of Turicibacter, Lactobacillus, Ruminococcus, Bacteroides, Ligilactobacillus, and Oscillibacter improved.

## 4. Discussion

Hypertension is a common and severe chronic disease that poses a serious threat to human health and is also the primary cause of the global burden of diseases [1]. ACEI can effectively treat hypertension in patients with renal hypertension, but the chemical synthesis of ACEI will bring in many adverse effects, so there is a growing interest in extracting more effective and safer ACEIs from dietary sources [37]. Probiotic-rich foods are currently dominating the market, and dairy products are widely regarded as the most effective carriers of probiotics [38]. Fermented milks have gained increasing recognition among the public due to their positive health effects [39]. It was reported that fermented milk showed antihypertensive effects, and the long-term consumption of fermented dairy products may reduce the risk of hypertension [40]. In this study, we evaluate the antihypertension effect of a fermented milk produced using the combined starter culture mixed with *L. plantarum* Y44 in the SHRs. The results indicate that the long-term oral administration of the fermented milk produced using the combined starter culture mixed with *L. plantarum* Y44 reduced the blood pressure, alleviated the hypertension-associated intestinal flora disorder, and increased the intestinal SCFA contents of the SHRs, which were attributed to the ACE-inhibitory peptides, such as AMKPWIQPK, GPVRGPFPII, LNVPGEIVE, NIPPLTQTPV, and YQEPVL, produced in the fermented milk.

In addition, studies had reported that the fermented milk from *Lactiplantibacillus plantarum* SR37-3 and SR61-2 showed an antihypertensive activity in the NOS inhibitor L-NAME-induced hypertensive rats [41]. It was reported that different starter culture strains have different proteolytic activities and proteolytic systems, and produce ACE-inhibitory peptides with different activities in fermented milk [42]. In addition, the heterogeneity between different strains leads to differences in their metabolites, ultimately leading to different antihypertensive potentials. In this study, the mixed milk fermented using the three strains of *L. plantarum* Y44, Y12, and Y16 combined with commercial starter cultures showed different effects on relieving hypertension in SHRs.

Oxidative stress is the main cause of inflammation and plays a crucial role in the development of hypertension and organ damage [43]. Mice lacking mitochondrial superoxide dismutase exhibit elevated levels of oxidative and inflammatory markers, leading to an increase in blood pressure [44]. Studies have shown that the main cause of microalbuminuria in hypertensive patients is oxidative stress, and excessive ROS can cause glomerular cell dysfunction and renal vascular endothelial damage [45]. The oral administration of the fermented milk produced using the combined starter culture mixed with *L. plantarum* Y44 significantly decreased the MDA content and increased the NO content and SOD activity in the SHRs, indicating that the intragastric administration of the fermented milk produced using the combined starter culture mixed with *L. plantarum* Y44 relieved oxidative stress in SHRs.

Research has shown that chronic kidney disease is usually caused by persistent hypertension, and the disorder of the RAS has an undeniable impact on the occurrence and development of hypertension [46,47]. An ACE-AngII-AT1 axis and an ACE2-Ang (1-7)-Mas axis make up the RAS. The excessive activation of the ACE regulatory axis and inhibition of the ACE 2 regulatory axis can lead to increased blood pressure and kidney damage [48]. Previous studies have reported that RAS diseases play an undeniable role in the occurrence and development of hypertension [49]. In this study, the contents of ACE and Ang II in the sera of SHRs were decreased by the fermented milk administration, and the contents of ACE 2 and Ang 1-7 in the sera of SHRs were significantly increased. The RAS was regulated at the gene level by upregulating the expression of ACE 2 and the MAS receptor and downregulating the expression of the ACE receptor, thereby controlling blood pressure. Many physiological effects of Ang (1-7) were mediated by Mas receptors. Xu et al. found that the overexpression of the ACE 2 gene in the brain of a hypertensive mouse model induced by a continuous infusion of Ang II could improve the baroreflex sensitivity and parasympathetic nerve activity and slow down the development of neurogenic hypertension [50]. The results of this study prove that the antihypertensive effect of ACE 2 was mediated by Ang (1-7)-Mas. In addition, the transfection of the ACE 2 gene could inhibit Ang II-induced oxidative stress, which could be reversed by Mas receptor antagonist A779. These results suggested that ACE-inhibiting peptides produced in the fermented milk containing *L. plantarum* Y44, such as AMKPWIQPK, GPVRGPFPII, LNVPGEIVE, NIPPLTQTPV, and YQEPVL, may be involved in the blood pressure regulation of the ACE 2-Ang (1-7)-Mas axis, which rebalance RAS signaling to regulate blood pressure by downregulating the ACE-Ang II-AT1 axis and upregulating the ACE 2-Ang (1-7)-Mas axis. The contents of ALT and AST could indicate the degree of liver cell damage. The changes in ALT and AST in the sera of the SHRs indicated that the fermented milk produced using the combined starter culture mixed with *L. plantarum* Y44 was safer than captopril.

Studies have shown that hypertension can affect the structure of gut microbiota, reduce the diversity of the gut microbiota, and reduce the number of beneficial bacteria [51]. The difference in the intestinal flora composition between hypertensive and normal rats was mainly due to the increase in the ratio of Firmicutes to Bacteroidetes (F/B) and the decrease in microbial diversity in the SHRs. In this study, the fermented milk containing *L. plantarum* Y44 may regulate the disruption of gut microbiota caused by hypertension at the phylum level. Previous studies have demonstrated a connection between hypertension and an intestinal microbiota imbalance and circulating SCFA levels [52]. An increase in the number of bacteria (Bacteroides, Firmicutes, Lactobacillaceae, Prevotellaceae, Lachnospiraceae, and Alloprevotella) that produce SCFAs (acetic acid, propionic acid, and butyric acid), might be one reason why the fermented milk lowers blood pressure [52]. At the same time, SCFAs could, through the regulation of T cells, promote Ang II to produce an effector response and improve the function of the immune system to achieve the effect of a lower blood pressure [35]. Lal et al. reported that SCFAs, particularly butyrate, could help regulate blood pressure by directly activating the vagus nerve’s afferent nerve fibers after being absorbed into the intestine’s mucosal lamina propria nerve terminals [53]. Moreover, Goswami et al. showed that SCFAs can significantly activate vagal afferent neurons by increasing phosphorylation, exhibiting the order of butyrate > propionate > acetate [54]. Additionally, butyrate can directly act on the central nervous system to regulate blood pressure by crossing the blood–brain barrier through specific transport proteins. In addition, the expression of butyric acid receptors in the hypothalamus of SHRs was relatively low, leading to a decrease in reactivity. Therefore, the role of butyrate in blood pressure regulation was affected. Some studies have found that the propionate SCFAs have a good antihypertensive effect [36]. Acetate also has a positive effect on regulating hypertension and preventing atherosclerosis, and malonate can improve cardiac function after myocardial infarction [55]. Similarly, Onyszkiewicz et al. demonstrated that butyrate can enter the bloodstream through the intestinal vascular barrier and act on GPR41 and GPR43 to relax the mesenteric arteries, thereby significantly reducing hypertension [56]. Therefore, we speculated that our fermented milk combined with *L. plantarum* Y44 can alleviate hypertension-associated intestinal microbiota dysbiosis by recovering the diversity of the gut microbiota and altering the key floras, which are SCFA producers, and that SCFA further reduces blood pressure and improves cardiovascular effects through G protein-coupled receptors.

## 5. Conclusions

In this study, we used the commercial starter combined with *L. plantarum* strains to ferment the mixed milk of cow milk and soy milk. Our results showed that the combined starter culture containing *L. plantarum* Y44 can generate ACE-inhibitory peptides in the fermented milk, such as AMKPWIQPK, GPVRGPFPII, LNVPGEIVE, NIPPLTQTPV, and YQEPVL, and its characteristics depend on the strain. And the long-term oral administration of the fermented milk can reduce blood pressure, alleviate the hypertension-associated intestinal flora disorder by restoring the intestinal flora diversity, and increase the intestinal SCFA levels in the SHRs. SCFAs can further reduce the blood pressure and improve the cardiovascular function through G protein-coupled receptors. The results indicate that the fermented milk from the combined starter culture containing *L. plantarum* Y44 can be used as a functional food with antihypertension effects.

## Figures and Tables

**Table 1:** Group information and administration of animal experiments.

Animals	Groups	Feeding
SHR	Positive control group (GY)	Captopril
SHR	Negative control group (KB)	Pasteurized milk
WKY	Blank group (WKY)	Pasteurized milk
SHR	Y12 group (Y12)	6038 + 6047 + Y12 fermented milk
SHR	Y44 group (Y44)	6038 + 6047 + Y44 fermented milk
SHR	Y16 group (Y16)	6038 + 6047 + Y16 fermented milk
The gavage dose was consistent among the groups, with a dosage of 10 mg/kg/d.		

GY: Gavage feeding of captopril to SHRs; KB: gavage feeding of pasteurized milk to SHRs; WKY: gavage feeding of pasteurized milk to WKYs; Y12: gavage feeding of the fermented milk produced using the combined starter culture mixed with *L. plantarum* Y12 to SHRs; Y44: gavage feeding of the fermented milk produced using the combined starter culture mixed with *L. plantarum* Y44 to SHRs; Y16: gavage feeding of the fermented milk produced using the combined starter culture mixed with *L. plantarum* Y16 to SHRs.

**Table 2:** Chemical characteristics of the fermented mixed milk with compound starter at 4 °C.

	Type of fermented milk	6038 + 6047	6038 + 6047 + Y12	6038 + 6047 + Y44	6038 + 6047 + Y16
Chemical characteristics					
TA (°T)		90.09 ± 3.09 ^b^	98.96 ± 2.27 ^a^	97.75 ± 5.91 ^a^	95.80 ± 1.77 ^a^
Viable count (CFU mL^−1^)		3.88 ± 0.52 × 10^9 b^	4.35 ±0.47 × 10^9 a^	4.59 ± 0.54 × 10^9 a^	4.54 ± 0.36 × 10^9 a^
Free amino acids (mg/mL)		0.303 ± 0.097 ^c^	0.390 ± 1.06 ^b^	0.417 ± 0.93 ^b^	0.493 ± 0.94 ^a^
Sensory score (points)		85.25 ± 3.39 ^c^	91.25 ± 4.11 ^a^	88.67 ± 3.17 ^b^	92.65 ± 2.47 ^a^
ACE I activity (%)		25.98 ± 1.19 ^c^	46.80 ± 2.10 ^ab^	53.56 ± 0.69 ^a^	42.98 ± 1.19 ^b^

Data represent the mean ± SD ^(a–c)^; mean values with different letters are significantly different (*p* < 0.05) according to the Duncan multiple range test.

**Table 3:** Peptide differential analysis between different fermented mixed milks.

Comparisons	All	Up	Down
Y vs. Y12	750	331	419
Y vs. Y44	982	374	608
Y vs. Y16	96	37	59
Y12 vs. Y44	887	511	376
Y12 vs. Y16	673	346	337
Y44 vs. Y16	886	371	443

Note: Y: 6038 + 6047; Y12: 6038 + 6047 + Y12; Y44: 6038 + 6047 + Y44; Y16: 6038 + 6047 + Y16.

**Table 4:** Peptides predicted to have ACE inhibitory ability.

Polypeptide Sequence	Mass	Length	*m*/*z*	Relative Content			
				Y	Y12	Y44	Y16
DAYPSGAW	1141.5079	8	571.7617	0.0429	0	0	0
EMPFPK	1007.4786	6	504.745	0.2865	0.1977	0	0
FVAPFPEVFG	1108.5593	10	555.2863	0.3365	0.4932	0.1881	0.6551
HLPLP	575.3431	5	288.6788	0.3818	0.2455	0	0
AMKPWIQPK	1097.6056	9	549.8098	9.1141	56.9335	57.9351	18.3578
DKIHPF	755.3966	6	378.7053	1	4.1244	2.9753	4.7795
EIVPNSAEERLH	1392.6997	12	465.2398	0	0	0.0714	0
FALPQY	737.3748	6	369.6942	2.4255	0	0	0.4849
FALPQYLK	978.5538	8	490.2833	0	0	0	0.1396
FPEVFGK	822.4276	7	412.2204	0.0138	0.0573	0	0
FVAPFPEVFG	1108.5593	10	555.2863	0.3365	0.3817	0.1881	0.6551
GPVRGPFPII	1051.6178	10	526.8159	1.9852	18.2349	68.8615	3.4358
IPPLTQTPV	964.5593	9	483.287	0	1.0812	6.5293	0
KAAAAP	726.4388	6	364.2252	0	0	0.0717	0
KYIPIQ	760.4483	6	381.2311	0	0.4559	0.8266	0
LDAQSAPLR	969.5243	9	485.77	0.7855	0	0	0.2509
LGPVRGPFP	938.5338	9	470.2735	0.4908	0.7913	0	0.2365
LHLPLPL	801.5112	7	401.7628	9.2085	1.8828	3.6409	1.8227
LNVPGEIVE	968.5178	9	485.2659	0.0144	0.6265	16.0615	0.8688
LTQTPVVVPPF	1196.6804	11	599.3472	6.9091	0	0	1.2390
LVYPFPGPIH	1138.6174	10	570.316	0	2.2781	0	0.1767
LVYPFPGPIPNSLPQN	1751.9246	16	876.968	0	0.0204	0.0696	0.1572
LVYPFPGPIPNSLPQNIPP	2059.1143	19	1030.5667	0.7600	0	0	0.6918
MKPWIQPK	1026.5685	8	514.2914	1.2883	8.5013	2.3931	1.8913
NIPPLTQTPV	1078.6023	10	540.308	5.0910	56.5832	76.8847	15.6471
NLHLPLP	802.4701	7	402.2415	0	4.5065	1.0877	0
PFPEVFGK	919.4803	8	460.7472	7.9391	6.6073	1.0813	13.6954
QEPVLGPVRGPFP	1391.7561	13	696.8857	37.6619	95.2655	1.9809	80.9390
TTMPLW	747.3625	6	374.6879	0	0.6130	0.1559	0
VPSERYL	862.4548	7	432.2333	0.0000	0.0983	0.5551	0.2403
VRGPFP	671.3755	6	336.6948	1.6800	0	0	0
VRGPFPIIV	996.612	9	499.313	1.7564	2.6911	0.6419	1.0535
VVVPPF	656.3897	6	329.2016	0.2691	0	0.2359	0
YAKPA	548.2958	5	275.1553	0	0	1	0
YIPIQY	795.4167	6	398.715	0	0.3886	0.5081	0
YPQRDMPIQ	1146.5492	9	574.2802	0	0	1	0
YQEPVL	747.3802	6	374.6969	0.2386	6.2676	5.2048	0.6514

Note: Y: 6038 + 6047; Y12: 6038 + 6047 + Y12; Y44: 6038 + 6047 + Y44; Y16: 6038 + 6047 + Y16; the content of the polypeptide is normalized to the median; that is, each column of data is divided by the median of the column of data.

## Data Availability

The original contributions presented in the study are included in the article, further inquiries can be directed to the corresponding author.

## Appendix A

**Table A1:** Sensory evaluation standard of compound fermented milk.

Evaluation Index	Evaluation Index	Score
Sour–sweet ratio (15)	Moderate sweet and sour	11–15
	Slightly sour or slightly sweet	6–10
	Bitter, too sour, or too sweet	0–5
Viscosity (15)	Medium viscosity, thick	11–15
	Viscosity is viscous or thin	6–10
	Viscosity is too sticky or too thin	0–5
Consistency (15)	Good viscosity, fine and silky	11–15
	Better viscosity and fineness	6–10
	Poor viscosity and no wire drawing	0–5
Taste (20)	No sense of graininess, smoothness and astringency	16–20
	The taste of the granules is slightly soft or hard and slightly powdery	11–15
	The texture of the granules is soft or hard and slightly powdery	6–10
	The taste of the granules is too soft or too hard, and the powder is very astringent	0–5
Flavor (20)	Fermented milk has an obvious flavor, natural fermentation flavor and smell, and no peculiar smell	16–20
	The flavor of the fermented milk is not enough, and the flavor of natural fermentation is low	11–15
	The flavor of the fermented milk is poor, and the flavor of natural fermentation is poor	6–10
	Fermented milk has no characteristic flavor or produces an unpleasant flavor	0–5
Texture (15)	Uniform color, milky white, good organization, no bubbles, no whey precipitation	11–15
	Light gray or grayish white color, good structure, slight whey precipitation	6–10
	Abnormal color, rough tissue and severe whey precipitation	0–5
